# The imaging appearances of various pericardial disorders

**DOI:** 10.1186/s13244-019-0728-4

**Published:** 2019-03-29

**Authors:** Emre Ünal, Musturay Karcaaltincaba, Erhan Akpinar, Orhan Macit Ariyurek

**Affiliations:** Department of Radiology, School of Medicine, Hacettepe University, 06100 Ankara, Turkey

**Keywords:** Pericardial patch, Hydatid disease, Pericardial metastases, Absence of the pericardium, Pericardial diverticulum, Pericardial mesothelioma, Gastropericardial fistula

## Abstract

The pericardium could be involved in a variety of clinical disorders. The imaging findings are not specific for an individual pathology in most of the cases; however, patient’s clinical history may guide radiologist to a definitive diagnosis. Congenital absence of the pericardium could be recognized with the imaging appearance of interposed lung tissue between the main pulmonary artery and aorta. Pericardial effusion is a non-specific condition that may occur due to inflammatory, infectious, and neoplastic disorders. Cardiac tamponade may occur in case of massive or rapid accumulation of fluid in the pericardial sac. Pericardial calcification is a common and easily identified entity on a computed tomography (CT) scan. Presence of calcification and/or fibrosis may result in pericardial constriction. Nevertheless, the pulsation of an adjacent coronary artery may prevent calcification formation in a focal area and consequently may result in pericardial diverticulum containing epicardial fat and coronary artery. The imaging findings encountered in patients with pericardial hydatid disease and Erdheim-Chester disease may mimic those of pericardial neoplasia. Pericardial adhesions and pedicled fat flaps may cause confusion on a CT scan in the post-surgical period following cardiac surgery. Pericardial fat necrosis can be diagnosed by CT in patients with chest pain. The radiologists should be familiar with the medical devices placed in pericardial space for certain individual indications. A pericardial patch and temporary epicardial pacemaker wires could be identified on a CT scan.

## Key points

- Imaging findings of various pericardial disorders are not specific for an individual pathology in most of the cases; however, patient’s clinical history may guide radiologist to a definitive diagnosis.
- Congenital absence of the pericardium could be recognized with levorotation of the heart and prominent main pulmonary artery.
- A pericardial patch or temporary epicardial pacing wires could be identified on a CT scan.
- In the setting of diffuse pericardial calcification, pulsation of an adjacent coronary artery may prevent calcification formation in a focal area and consequently may result in pericardial diverticulum containing epicardial fat and coronary artery.

## Introduction

The pericardium could be involved in a variety of benign and malignant disorders. Plain radiography has limited value since pericardial disorders may not be differentiated from various types of mediastinal and/or cardiac pathologies on roentgenograms. Nevertheless, in case of pneumopericardium, plain radiography may give the definite diagnosis yet may not reveal the underlying cause. Computed tomography (CT) is the most widely used modality of choice for the evaluation of the pericardium; however, ultrasound could be easily and rapidly performed to reveal the presence of pericardial effusion which is a crucial finding particularly in trauma patients [1]. Pericardial calcifications, extension of pericardial collections and tumors, pneumopericardium and its underlying cause, pericardial/epipericardial fat necrosis, foreign bodies, and medical devices placed in the pericardial space can be revealed by CT [2–5]. However, soft tissue infiltration of pericardium could not be differentiated from accompanied pericardial effusion by CT in rare cases such as in Erdheim-Chester disease or inflammatory constrictive pericarditis. In this case, magnetic resonance imaging (MRI) can replace CT due to its superior soft tissue contrast resolution (Table 1) [6, 7].

**Table 1:** The role of imaging modalities in various pericardial disorders

		Roentgenogram	CT	MRI
Absence of the pericardium	Interposed lung tissue	++	+++	+++
	Diminished right cardiac border	++	*	*
	Levorotation of heart	+	+++	+++
	Pericardial discontinuity	–	++	+++
Pneumopericardium	Imaging-based diagnosis	+	+++	**
	Revealing the underlying cause	–	+++	**
Pericardial fluid collection	Imaging-based diagnosis	+	+++	+++
	Discrimination of fluid content	–	++	+++
	Revealing the underlying cause	+	++	++
	Revealing the pericardial thickening	–	++	+++
Erdheim-Chester disease	Discrimination of involvement	–	++	+++
Pericardial calcification	Revealing the burden of involvement	+	+++	++
	Revealing the compressive effect to heart chambers	+	+++	+++
Pericardial masses	Discrimination of cystic/solid/fat/calcific content	–	++	+++
Hydatid disease	Imaging-based diagnosis	–	++	+++
	Revealing the association between cardiac chambers	–	++	+++
	Revealing the presence of daughter cysts/floated membrane	–	++	+++
Medical devices	Drainage catheters	++	+++	+++
	Temporary epicardial pacing	++	+++	***
	Pericardial patch	–	++^a^	++^a^
Fat necrosis	Imaging-based diagnosis	–	++^b^	+++^c^
Foreign body	Imaging-based diagnosis	+	+++	+^a^

^*^Apparent on coronal view

^**^MRI is usually not performed due to need for urgent intervention

^***^May induce artifact

^a^Depends on the substance

^b^Subtle form of inflammation could be overlooked on CT

^c^Findings may vary in correlation with the stages of fat necrosis

In this article, we will review the various types and causes of pericardial disorders with emphasis on cross-sectional imaging findings.

## Normal pericardium anatomy

The pericardium is seen as a linear line (< 2 mm) covering the heart and also the roots of the great vessels (proximal portions of the ascending aorta, pulmonary artery, left pulmonary veins, and superior vena cava) on CT or MRI images [6, 8, 9]. The pericardium consists of outer fibrous and inner serous layers. The serous part has an outer parietal and inner visceral layers. The pericardial space lies between the parietal and visceral parts of the serous layer and contains 15–50 mL of serous fluid produced by visceral pericardium (plasma ultrafiltrate and cardiac lymph) [5]. The parietal layer of serous pericardium lines the fibrous pericardium, and the visceral layer covers the epicardial surface of the heart and great vessels. The fibrous layer is continuous with the diaphragm (pericardiophrenic ligament), sternum (sternopericardial ligaments), costal cartilages, and external layer of the great vessels [5, 6, 8, 9].

### Absence of the pericardium

#### Congenital

**Fig. 1:**
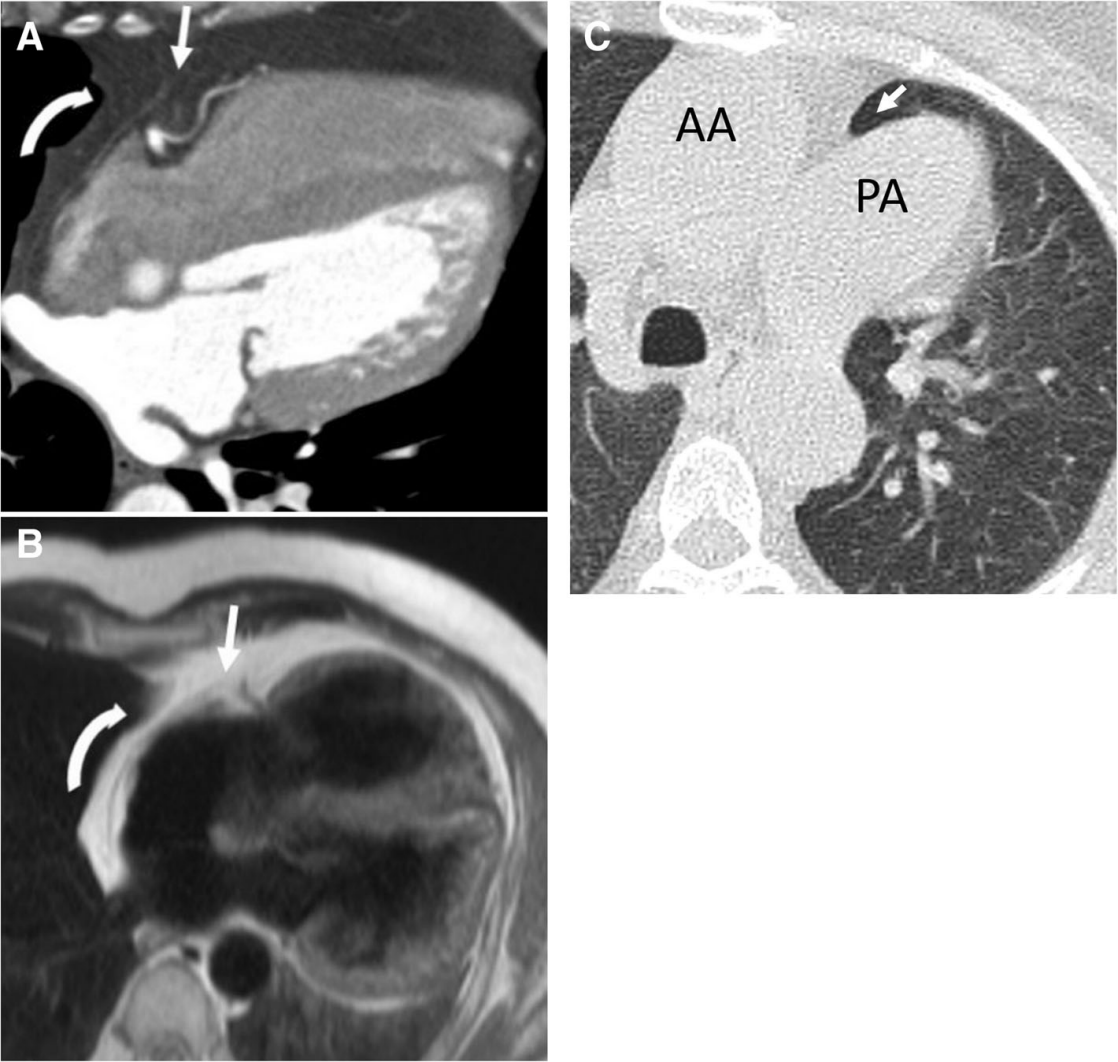
A 51-year-old man. Axial contrast-enhanced CT (**a**) and T2-weighted MR images (**b**) demonstrate levorotation (curved arrows) of the heart and lack of pericardial continuity (arrows). **c** Axial CT image of a different patient with congenital absence of the pericardium demonstrates a prominent main pulmonary artery (PA). Interposed lung tissue (arrow) between the main pulmonary artery and the ascending aorta (AA)

Absence of the pericardium is a rarely encountered malformation in clinical practice [6, 10, 11]. Its total prevalence still remains unknown [11]. A significant portion (30–50%) of the reported cases with absence of the pericardium were associated with congenital anomalies of the heart, lungs, chest wall, and diaphragm [10]. The absence is typically partial and occurs more commonly on the left side compared to the right or inferior aspects (Fig. 1) [6, 8, 12]. Premature atrophy of the left common cardinal vein which is the major source of blood supply to the left pleuropericardial membranes is the main reason for the congenital absence of the pericardium [5, 9, 10]. Patients are usually asymptomatic; however, serious complications may occur in case of left atrial appendage herniation which could be complicated with left coronary artery herniation and myocardial ischemia [5]. Lack of pericardial coverage at the aortopulmonary window creates a potential space resulting in the interposition of lung tissue between the main pulmonary artery and the aorta (Fig. 1). A prominent main pulmonary artery and levorotation of the heart are frequently encountered on CT and MRI (Fig. 1).

#### Acquired defect

**Fig. 2:**
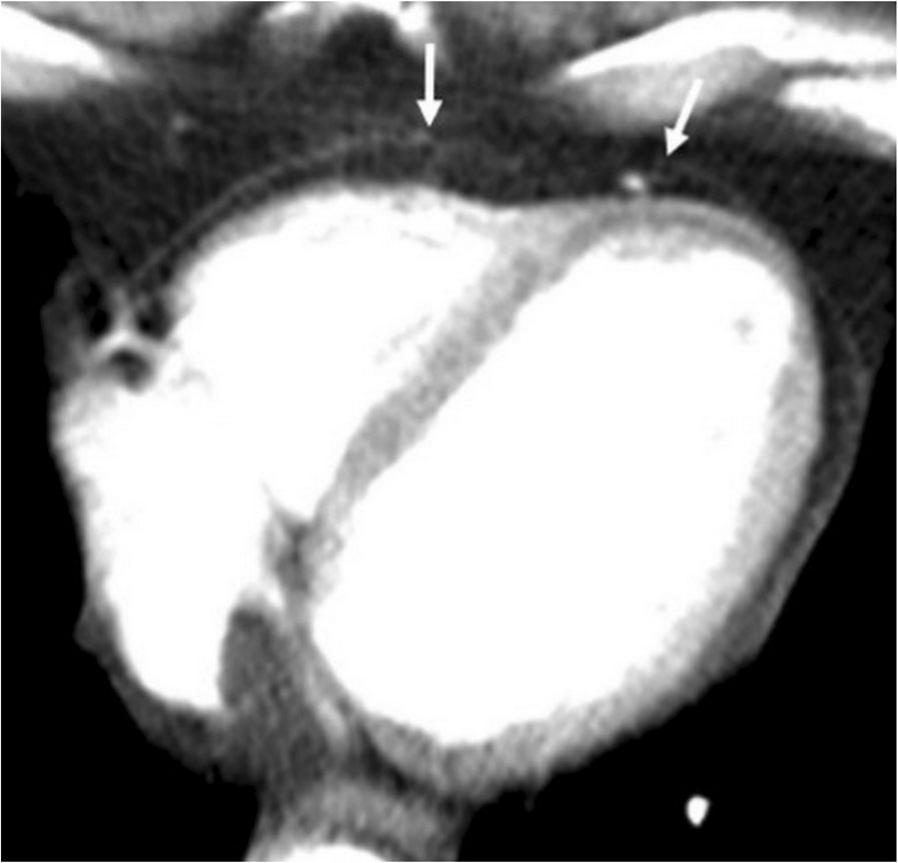
A 68-year-old man with previous coronary artery bypass surgery. Axial contrast-enhanced CT image demonstrates the lack of pericardial continuity (arrows) due to surgery

In addition to congenital etiology, the acquired defect of the pericardium could also be seen particularly following pericardial surgery (Fig. 2) [5]. Pericardial resection is performed either to relieve the compressive effect of constrictive pericarditis or to be able to reach the coronary arteries during coronary artery bypass grafting surgery. The imaging signs that were previously mentioned in the congenital absence of the pericardium are not observed in patients with pericardial resection.

### Pericardial collections

#### Pneumopericardium

**Fig. 3:**
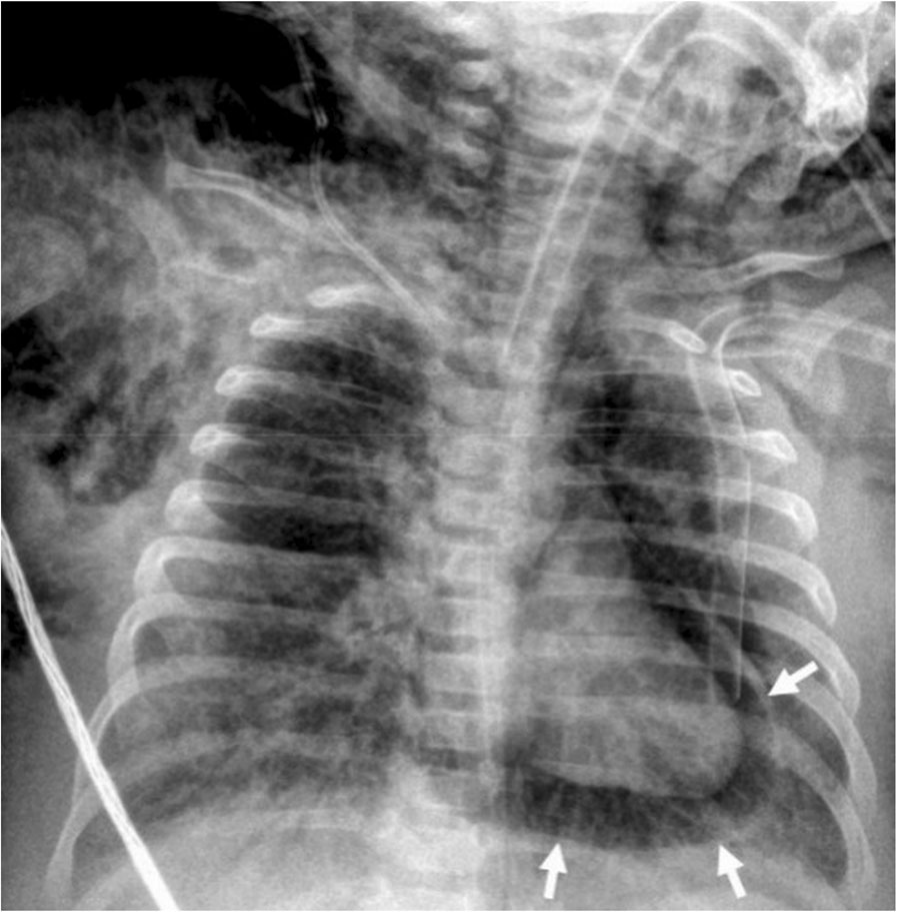
A 3-month-old girl with pneumonia and sepsis developed pneumopericardium (arrows) under endotracheal intubation at insensitive care unit

**Fig. 4:**
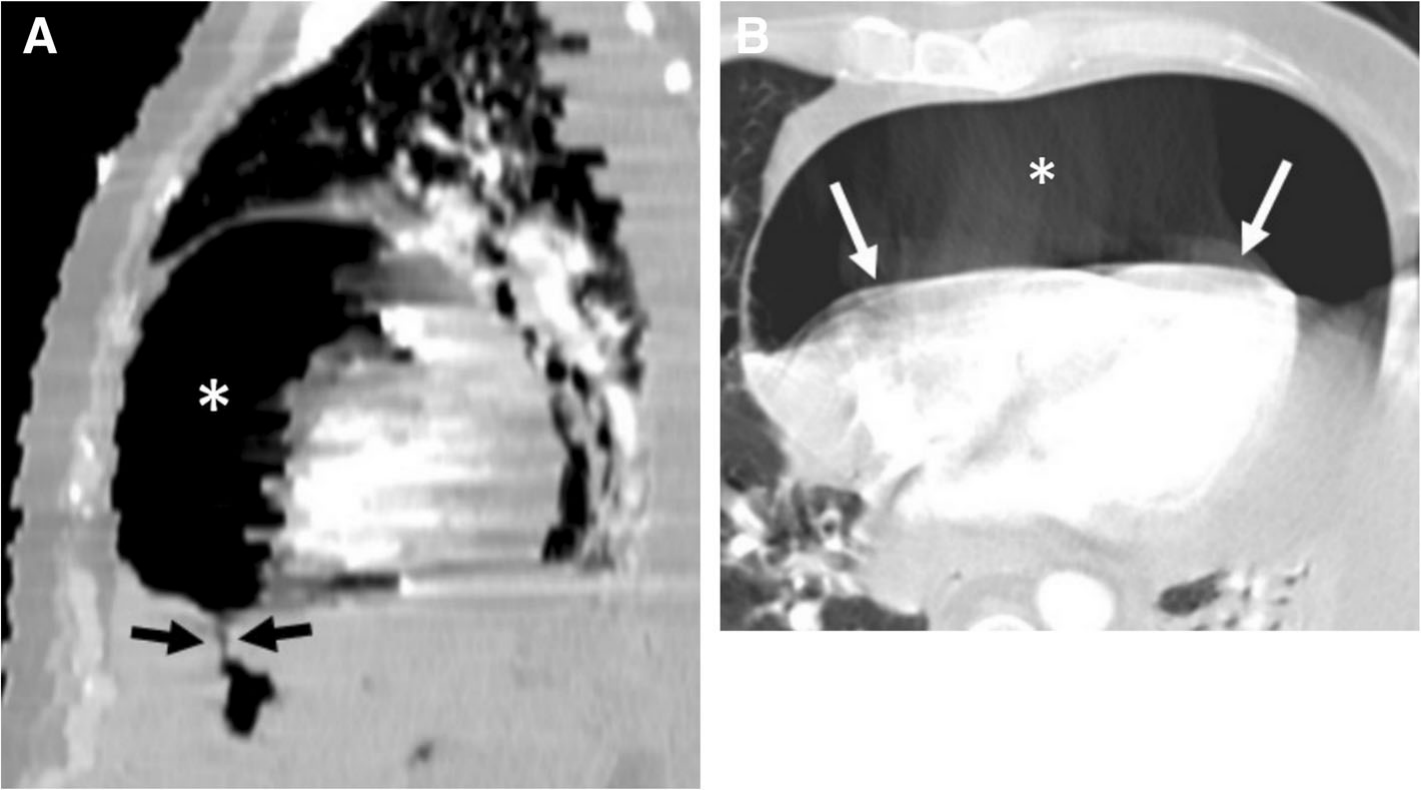
A 51-year-old man with pathologically proven gastric lymphoma developed sudden onset of dyspnea and cardiac arrhythmia. Contrast-enhanced CT in the sagittal (**a**) and axial (**b**) planes demonstrates a gastropericardial fistula (black arrows) complicated with massive pneumopericardium (asterisks). Significant compressive effect to the heart is also noted (white arrows)

Among the various causes of pneumopericardium, trauma is the most frequently encountered etiology [5, 8]. Pneumothorax or pneumomediastinum may or may not accompany to pneumopericardium. Intubated patients, particularly the pediatric population, are also at risk for the development of pneumopericardium due to positive pressure ventilation complicated with barotrauma (Fig. 3). The clinical symptoms may range from subtle chest pain to acute heart failure. The adjacent air-containing structures could also be questioned for the occurrence of pneumopericardium in the non-traumatic setting. Cardiac tamponade may occur in case of tension pneumocardium as a consequence of direct communication of pericardial space with the gastrointestinal tract. The diagnosis could be made by roentgenograms. However, CT may reveal the underlying cause (Fig. 4). MRI is not practical since patients with pneumopericardium usually need emergent care.

#### Pericardial fluid collections

**Fig. 5:**
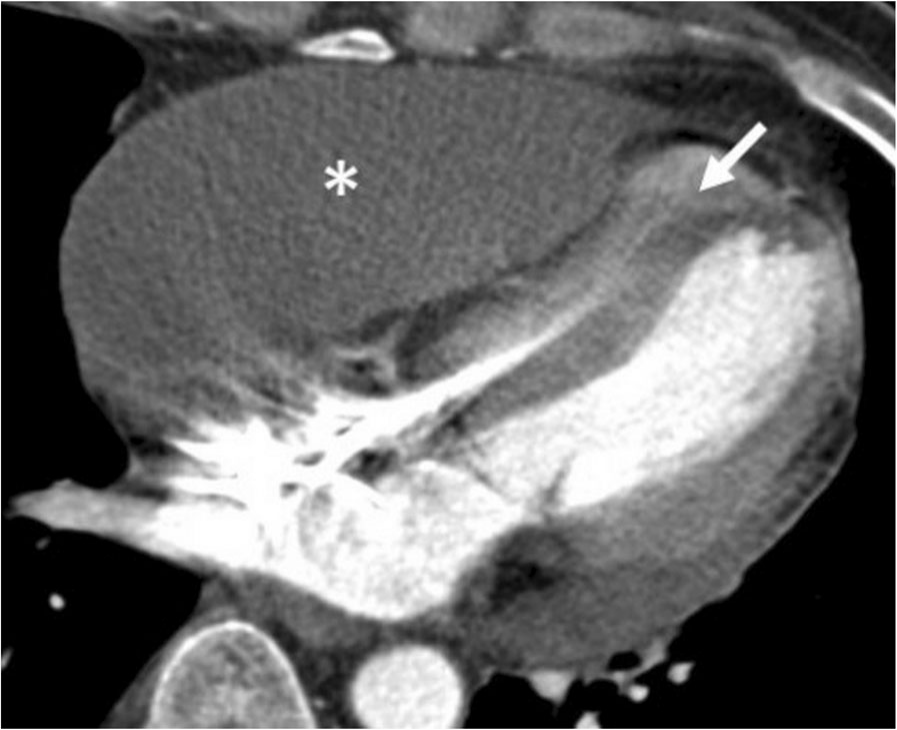
A 61-year-old man with right heart failure symptoms underwent to CT scan to rule out the presence of pulmonary artery embolism. Axial contrast-enhanced CT image shows significantly compressed right ventricle (arrow) due to the loculated pericardial effusion (asterisk)

Pericardial effusion is a frequently encountered disorder. Increased venous or lymphatic pressure is the common etiology of simple pericardial effusion; however, rheumatologic diseases, infection, malignancy, and trauma may also cause pericardial effusion [5, 8, 13]. Pericardial thickening could be associated with pericardial effusion. The differentiation of benign and malignant pericardial effusions by imaging could be challenging [3]. Echocardiography is the first-line imaging technique in the evaluation of pericardial effusions. [3, 14, 15]. However, there could be limitations in the detection of fluid due to acoustic windows and in cases of loculated fluid (Fig. 5). MRI is more sensitive than echocardiography in the detection of small collections, mostly in loculated fluid [15]. CT may also demonstrate the extension of effusion. Post-contrast images, particularly fat-saturated T1-weighted MR images, may reveal the presence of pericardial thickening. The contents of pericardial effusion either sampled or diagnosed by imaging (i.e., hemorrhagic, serous, or pyogenic) may give clue for the etiology.

##### Hemorrhagic collections

**Fig. 6:**
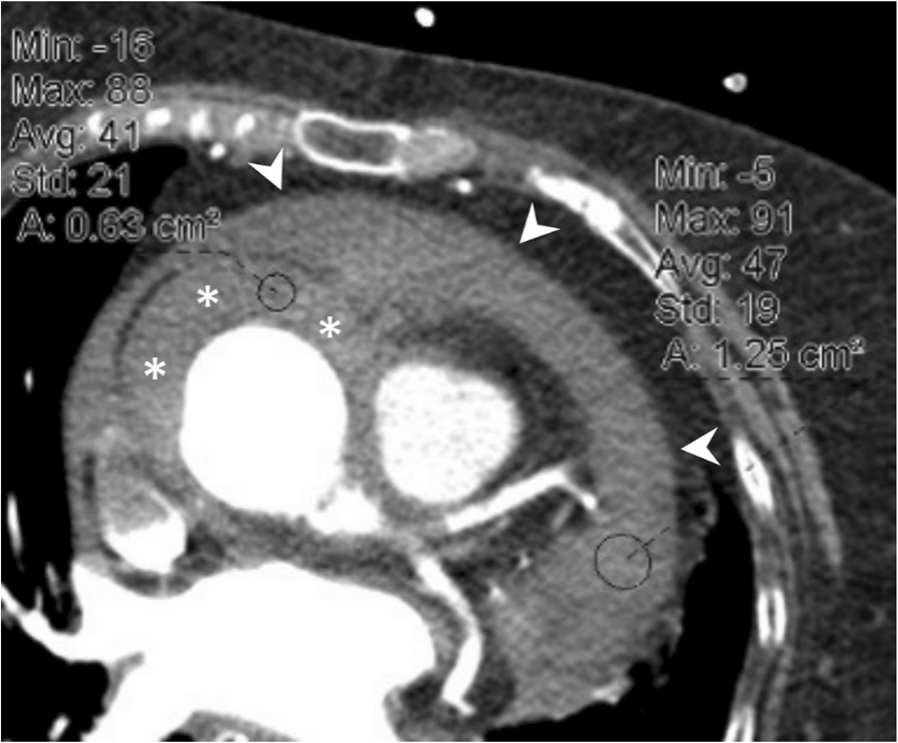
A 86-year-old woman with acute onset of pain in the chest. Axial contrast-enhanced CT image reveals hemopericardium (arrowheads) and intramural hematoma of the ascending aorta (asterisks)

**Fig. 7:**
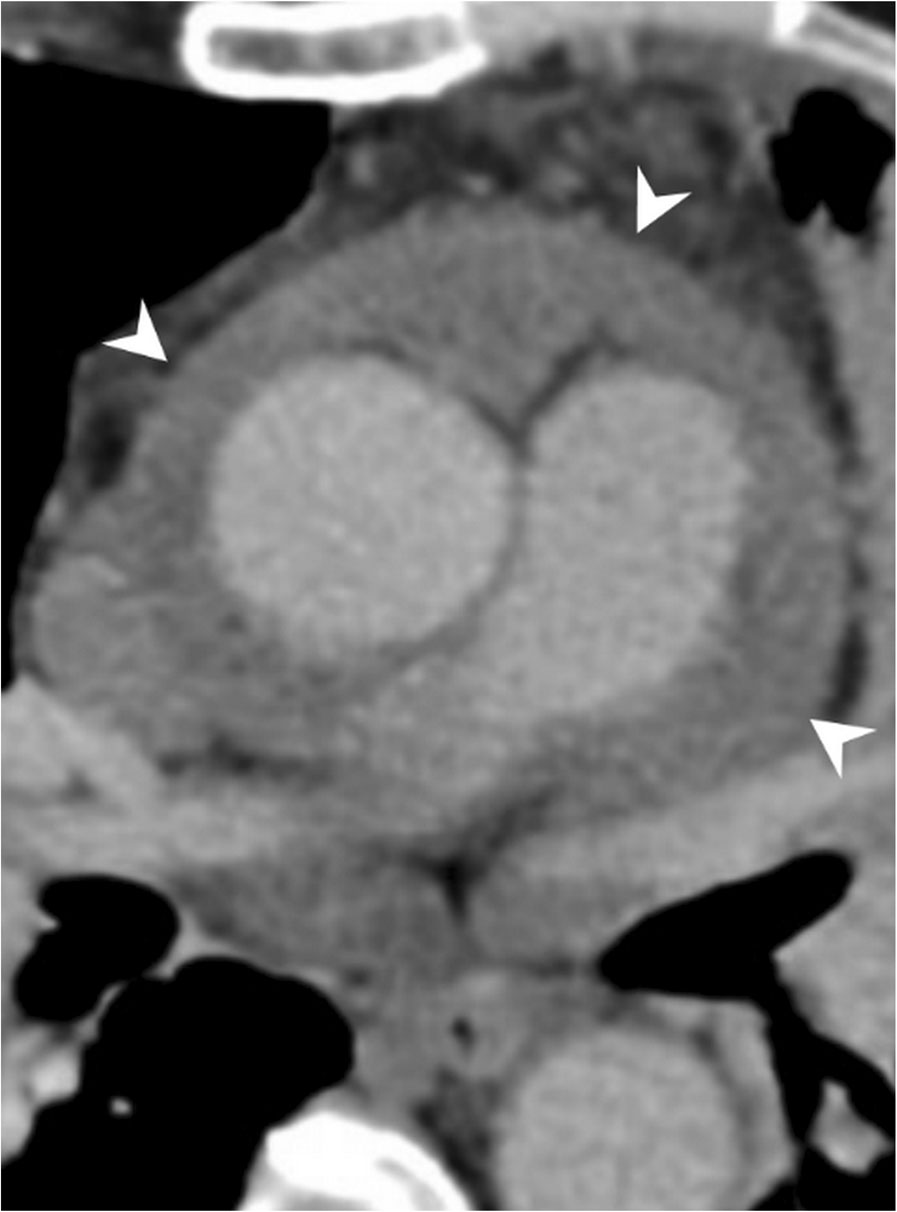
A 52-year-old woman with breast cancer receiving radiotherapy for treatment. Axial contrast-enhanced CT image reveals pericardial effusion with increased density compatible with hemopericardium (arrowheads)

Pericardial hemorrhage can be seen as an unfortunate complication of the ascending aorta aneurysm or trauma (Fig. 6) [16]. Myocardial infarction, iatrogenic injury during surgery, anticoagulant therapy, and malignancy may also result in pericardial hemorrhage. In addition, radiotherapy (RT) may also induce hemorrhagic pericardial effusion and thickening [6, 8]. Patients with lymphoma and breast and esophageal cancers are potential candidates for RT-induced hemorrhagic pericardial effusion and other pericardial disorders (Fig. 7). Patients may present with acute chest pain. In case of massive or rapid accumulation of blood, cardiac tamponade and heart failure may occur [16]. Ultrasound may reveal the presence of pericardial effusion; however, the differentiation of pericardial hemorrhage from simple pericardial effusion could be challenging based on ultrasound findings. Nevertheless, CT may demonstrate increased density of hemorrhagic collection and also may give clues for the etiology of pericardial hemorrhage. Although the signal intensity of hemorrhage may vary depending on its age, typically high signal intensity on T1-weighted MR images often indicates the presence of hemorrhage [6].

##### Non-hemorrhagic fluid collections

**Fig. 8:**
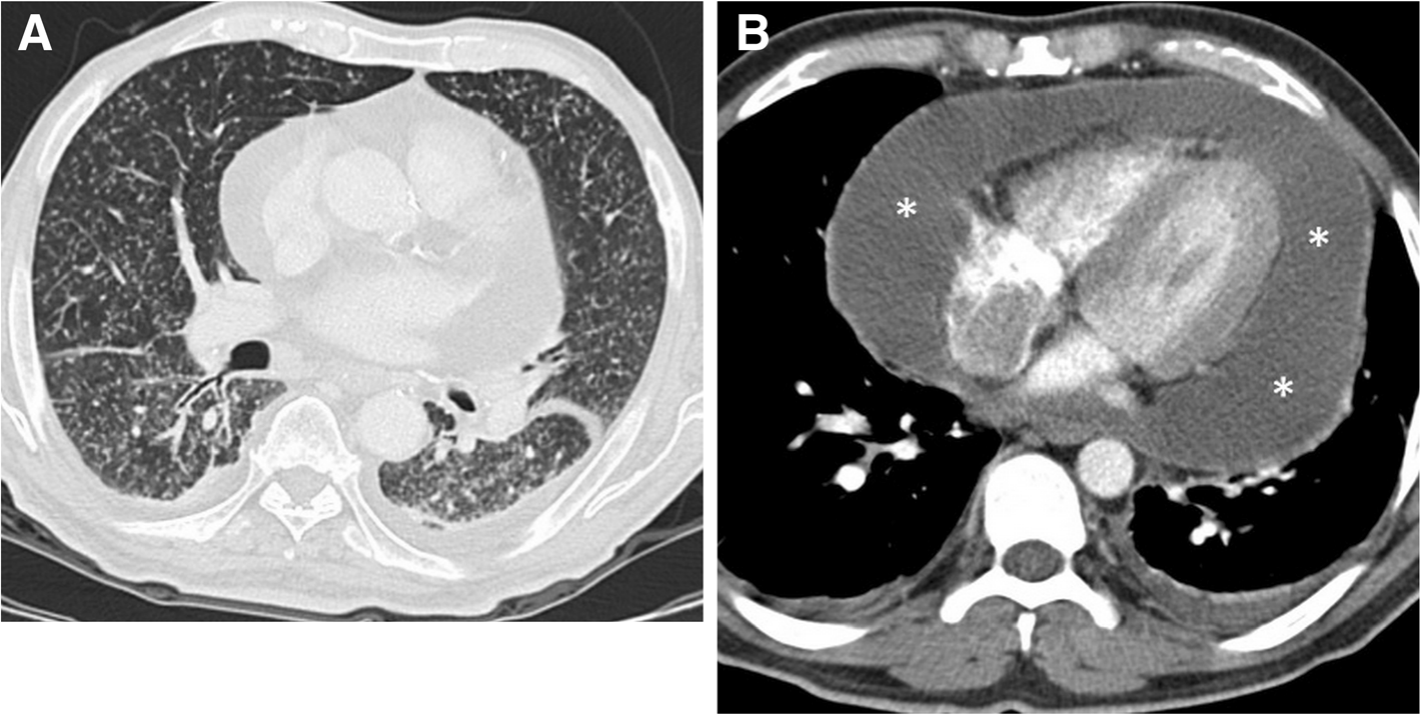
A 41-year-old man with newly diagnosed pulmonary tuberculosis. **a** Axial contrast-enhanced CT image at lung window reveals miliary pattern of pulmonary involvement. **b** Large amount of pericardial effusion (asterisks) is also noted. A culture test was positive for tuberculosis following the US-guided pericardiocentesis

Pericardial non-hemorrhagic fluid collections have a wide range of differential diagnosis including systemic lupus erythematosus, rheumatoid arthritis, Sjogren’s syndrome, dermatomyositis, acute viral or bacterial infections, tuberculosis, cardiac and renal failure (uremia), radiation, surgery, hypothyroidism, malignancy, myocardial infarction, and trauma [3, 5, 6]. Nevertheless, the imaging findings are non-specific and previous clinical history is important for most of the cases with pericardial effusion. Among the causes of infectious pericardial collections, viral pathogens are more frequently seen [14, 15, 17]. Conservative treatment is usually adequate for viral pericarditis. In contrast to other infectious agents, tuberculous (TB) pericarditis could be diagnosed by imaging. The presence of miliary pattern of pulmonary parenchymal involvement should raise a suspicion for TB pericarditis (Fig. 8). However, in patients with HIV infection, TB may cause more aggressive form of involvement including mediastinal granulomatous lymphadenitis and mediastinitis, due to the decreased immune response of the host. Despite the underlying cause, early diagnosis and treatment of pericardial effusion is important since untreated pericardial effusion or thickening could be complicated with pericardial calcification and constrictive pericarditis.

### Erdheim-Chester disease

**Fig. 9:**
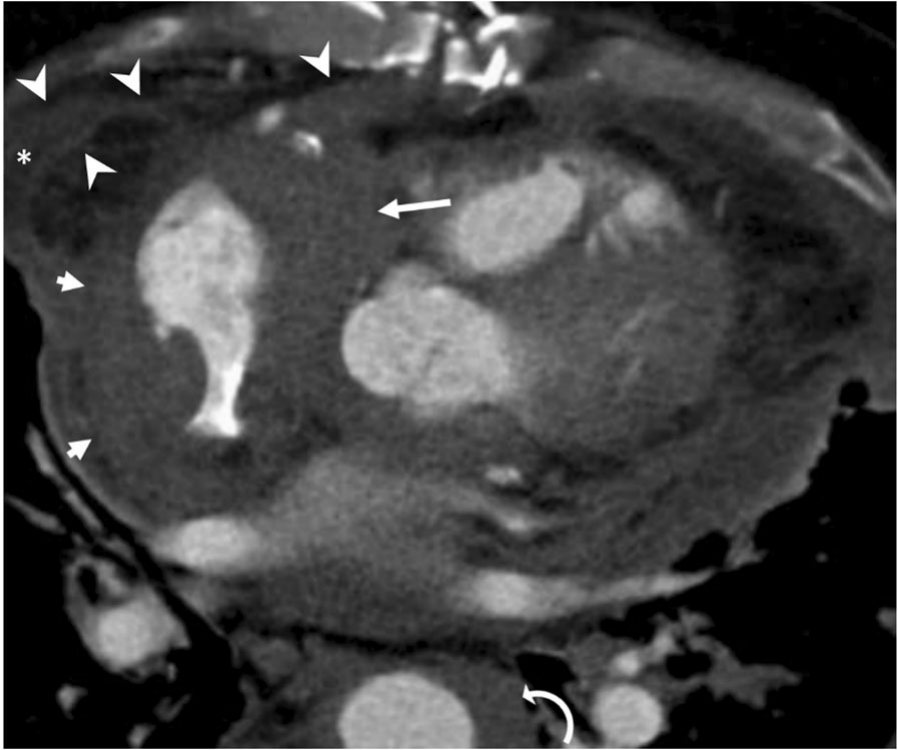
A 61-year-old man with Erdheim-Chester disease. Axial reformatted contrast-enhanced CT image demonstrates lobulated soft tissue densities with pseudo-tumor appearance involving the right atrium (short arrows), right atrioventricular groove (long arrow), and the aortic wall (curved arrow). Pericardial (asterisk) effusion and thickening (arrowheads) are also seen

Erdheim-Chester disease (ECD) is a rare multisystem non-Langerhans cell histiocytosis that could be presented with various radiological signs [7, 18, 19]. Pericardial involvement may rarely be encountered in patients with ECD. Presenting symptoms may vary depending on the dissemination of the disease. Pericardial thickening and effusion are the common imaging findings. Pericardium is commonly infiltrated with mass-like soft tissue lesions, and imaging appearances may mimic those of the loculated pericardial effusions on CT (Fig. 9). However, MRI may better differentiate the pericardial soft tissue infiltration from pericardial effusion [7, 18]. Infiltration of the epicardium and/or myocardium is frequently encountered in the right atrium and atrioventricular groove with pseudo-tumor appearance [7, 18, 19].

### Pericardial calcification

**Fig. 10:**
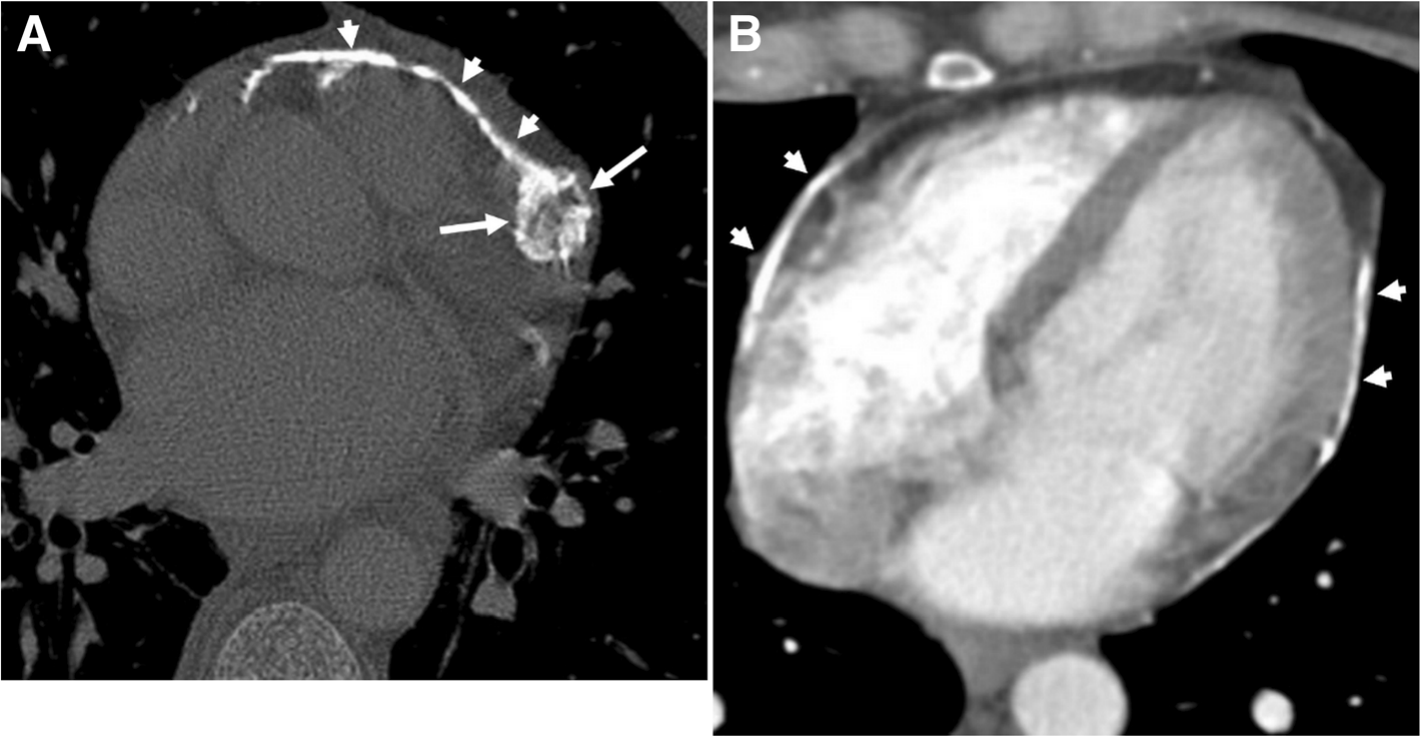
**a**, **b** Axial CT images of two different patients demonstrate the nodular (long arrows) and linear (short arrows) areas of calcification through the course of pericardium

**Fig. 11:**
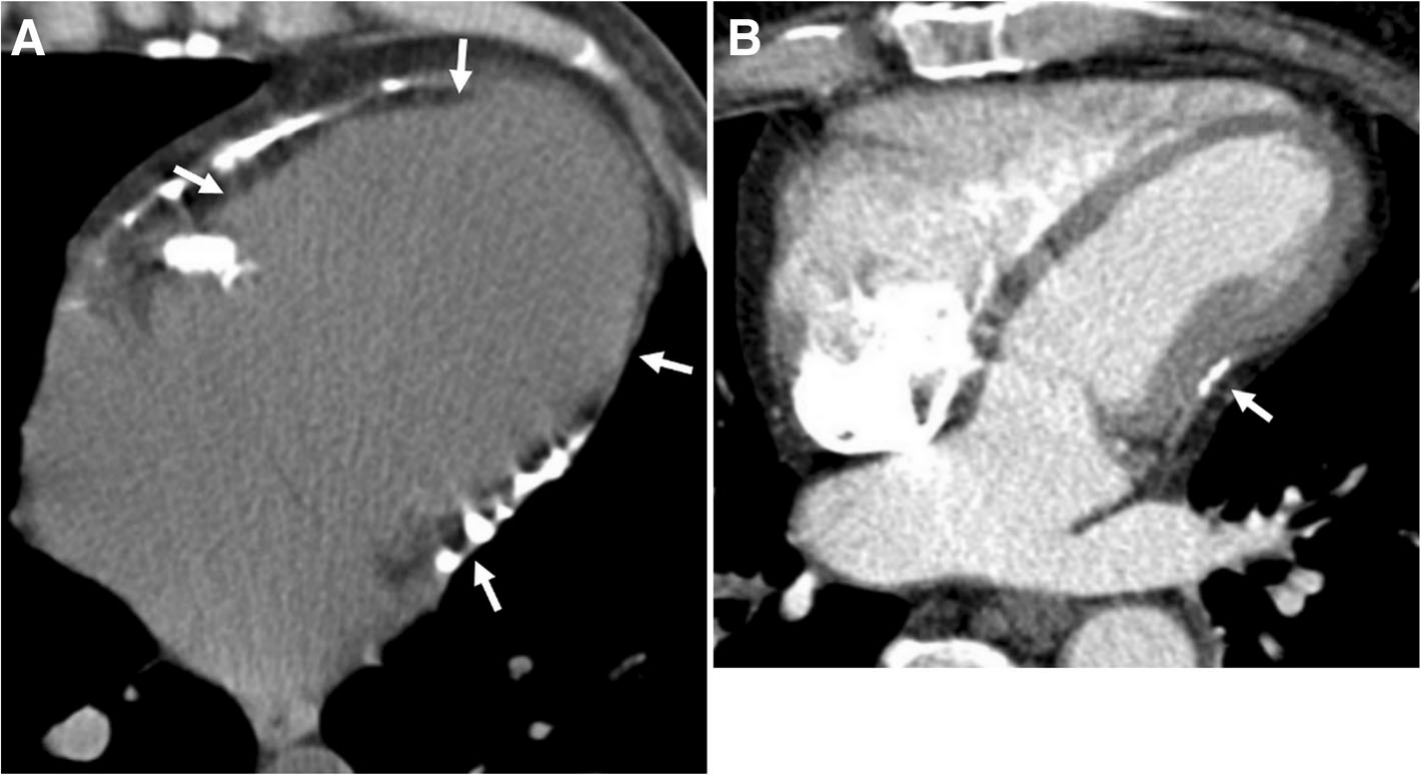
A 35-year-old man underwent to CT scan due to decreased cardiac output. **a** Axial CT image reveals entrapped heart appearance (arrows) with diffuse pericardial calcification which is compatible with constrictive pericarditis. **b** Axial contrast-enhanced CT image following surgery demonstrates diminished compressive effect to the right side of the heart; however, residual compressive effect to the left ventricle (arrow) was still evident despite surgery

**Fig. 12:**
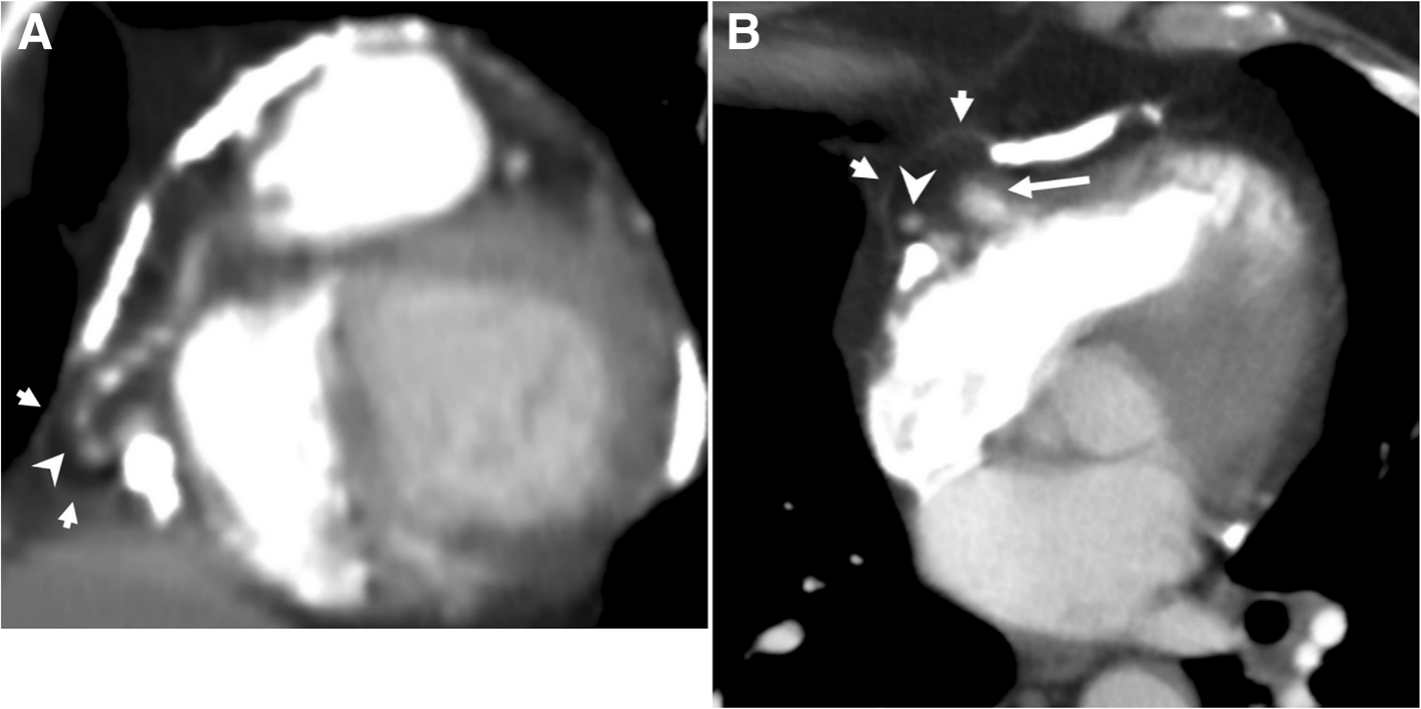
62-year-old woman with echocardiography findings suggestive of restrictive cardiomyopathy or constrictive pericarditis. Contrast-enhanced CT images on reformatted coronal (**a**) and axial plane (**b**) demonstrate linear pattern of extensive pericardial calcification. A pericardial diverticulum (short arrows) containing epicardial fat, coronary artery (arrowheads) and lateral wall of the right ventricle (long arrow) is noted

Pericardial calcification is a significant cause of pericardial constriction [17]. However, pericardial constrictions may also occur without calcification and even without accompanying pericardial thickening [9, 17, 20]. Pericardial calcification can be seen following various conditions including chronic pericarditis, tuberculosis, uremia, hemopericardium, radiation, idiopathic pericarditis, and surgery [5, 8]. Pericardial calcification does not always result in constrictive pericarditis and low cardiac output. CT is superior to MRI in the evaluation of pericardial calcifications; however, imaging appearances and pattern of involvement may vary among patients (Fig. 10). The calcification commonly occurs over the anterior and diaphragmatic aspects of the heart [21]. The atrioventricular grooves (more common in right) and right atrium are also frequently involved [17, 20, 21]. Higher myocardial contractibility and increased pressure in the left side of the heart could be the reason for the decreased rate of pericardial calcification in these areas. The presence of pericardial calcification adjacent to the left ventricle may indicate severe form of involvement. Surgical removal is preferred in case of increased systemic venous pressures and low cardiac output (Fig. 11). However, the compressive effect of residual lesions to heart chambers may persist even after surgery (Fig. 11). On the other hand, in the setting of diffuse pericardial calcification, pulsation of an adjacent coronary artery may prevent calcification formation in a focal area and consequently may result in pericardial diverticulum containing epicardial fat and the coronary artery (Fig. 12).

### Pericardial masses

#### Pericardial cyst

**Fig. 13:**
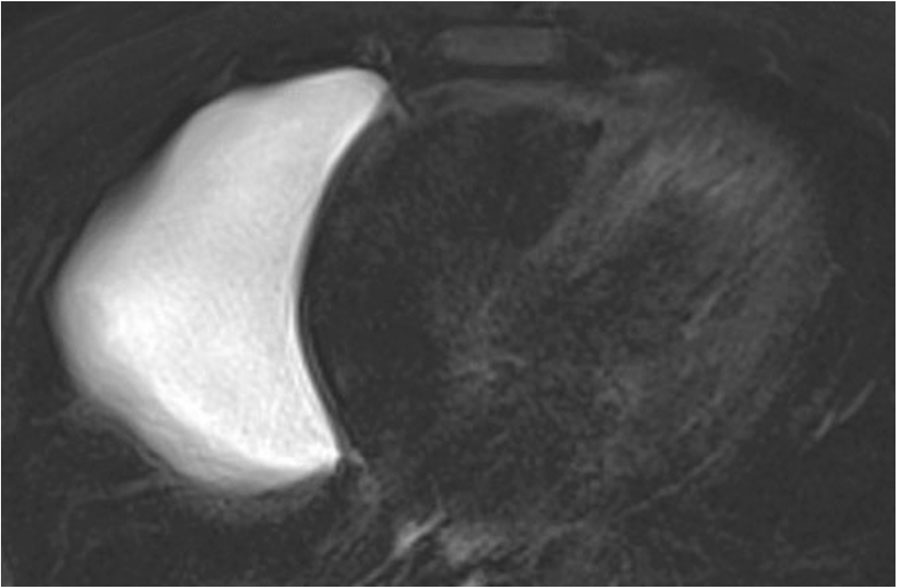
A 40-year-old man with incidentally detected pericardial cyst. Axial fat-suppressed T2-weighted MR image demonstrates hyperintense cystic mass in the right anterior cardiophrenic angle compatible with a pericardial cyst

The most common benign pericardial mass is a pericardial cyst, followed by lipoma [22]. A pericardial cyst is a congenital malformation that is caused by pinching-off of a parietal pericardial recess which turns into an isolated cyst [6, 9, 20]. Patients are usually asymptomatic but could present with symptoms related to compression in cases of larger pericardial cysts (Fig. 13). The most common location is the anterior cardiophrenic angle (right more common than left), but could be found anywhere along the pericardium. Pericardial cysts are well-defined, non-enhancing, homogeneous lesions demonstrating fluid attenuation on CT scans [22]. Low signal intensity on T1-weighted images and high signal intensity on T2-weighted images are common findings of pericardial cysts. However, some of the cysts may contain debris or hemorrhagic content that may appear hyperdense on CT and demonstrates high signal intensity with T1-weighted sequences and intermediate to low signal intensity with T2-weighted sequences [22]. In this setting, diffusion-weighted imaging could be used as a complementary tool for the diagnosis, since pericardial cysts do not demonstrate restricted diffusion [22, 23]. Differential diagnosis includes bronchogenic cyst and thymic cyst [22, 24]. A pericardial diverticulum may mimic the imaging features of a pericardial cyst; however, communication with the pericardial space is the distinguishing feature of a pericardial diverticulum [13, 20].

#### Primary tumors

**Fig. 14:**
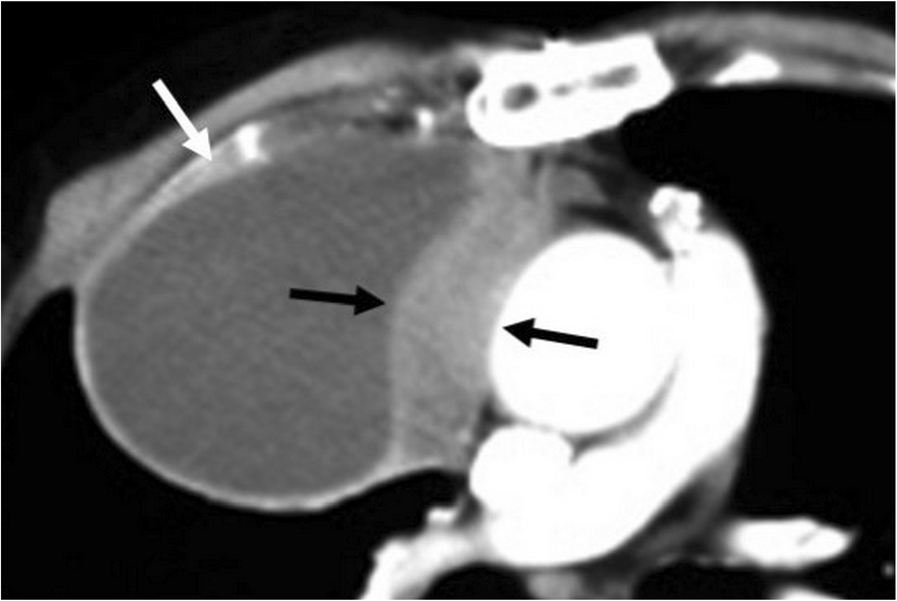
A 45-year-old man with pathologically proven pericardial mesothelioma. Axial contrast-enhanced CT image shows a heterogeneous pericardial mass with solid (black arrows) and cystic (white arrow) components. Note that there is a lack of fat tissue between the ascending aorta and the mass raising suspicion for perivascular invasion

Pericardial metastases are more frequently encountered than primary pericardial tumors [6, 9, 20, 22]. Primary pericardial tumors are rare entities in routine practice [6, 9, 20, 22]. The most common is mesothelioma followed by different sarcomas, lymphomas, and primitive neuroectodermal tumor [9, 22]. Symptoms and imaging signs are usually non-specific for an individual type of tumor. Mesothelioma could be presented with various imaging appearances on CT and MRI. Cystic and solid components could be encountered on CT (Fig. 14). MRI better demonstrates the distinction of solid parts within the mass. Lipoma, lipoblastoma, and liposarcoma could be characterized by fatty nature [22]. MR images, particularly fat-saturated sequences, may better demonstrate the fatty content of the mass. Pericardial effusion and/or thickening may accompany with mesothelioma or any other pericardial malignancies.

#### Secondary tumors

**Fig. 15:**
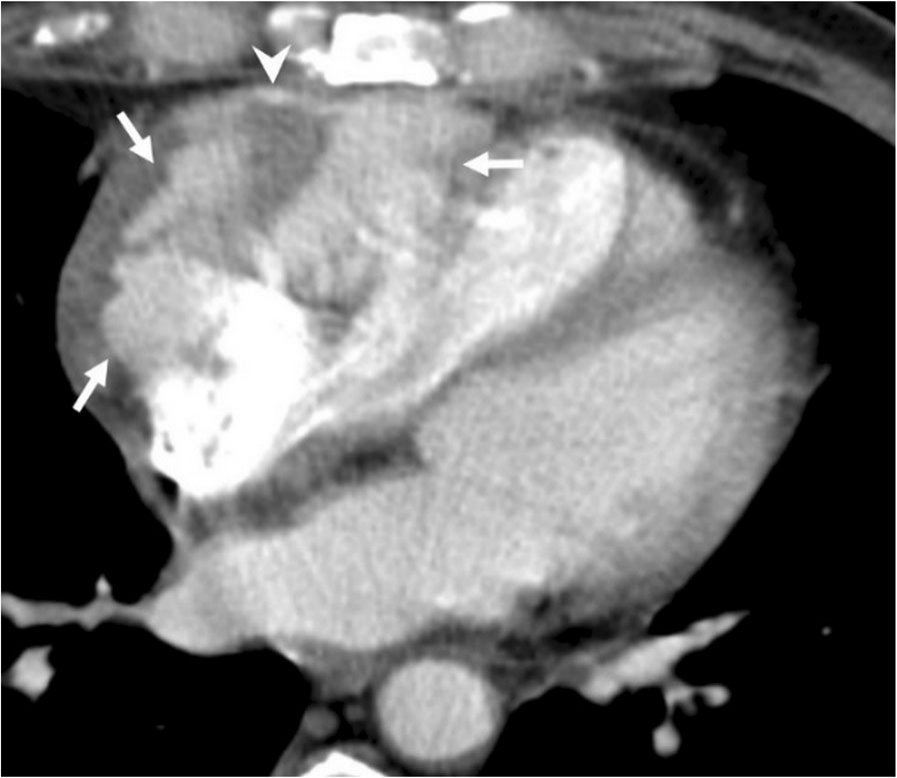
A 59-year-old man with pathologically proven primary cardiac angiosarcoma. Axial contrast-enhanced CT image reveals a highly vascular mass (arrows) invading the right atrioventricular groove, right atrium, and ventricle. Pericardial involvement (arrowhead) is also noted

**Fig. 16:**
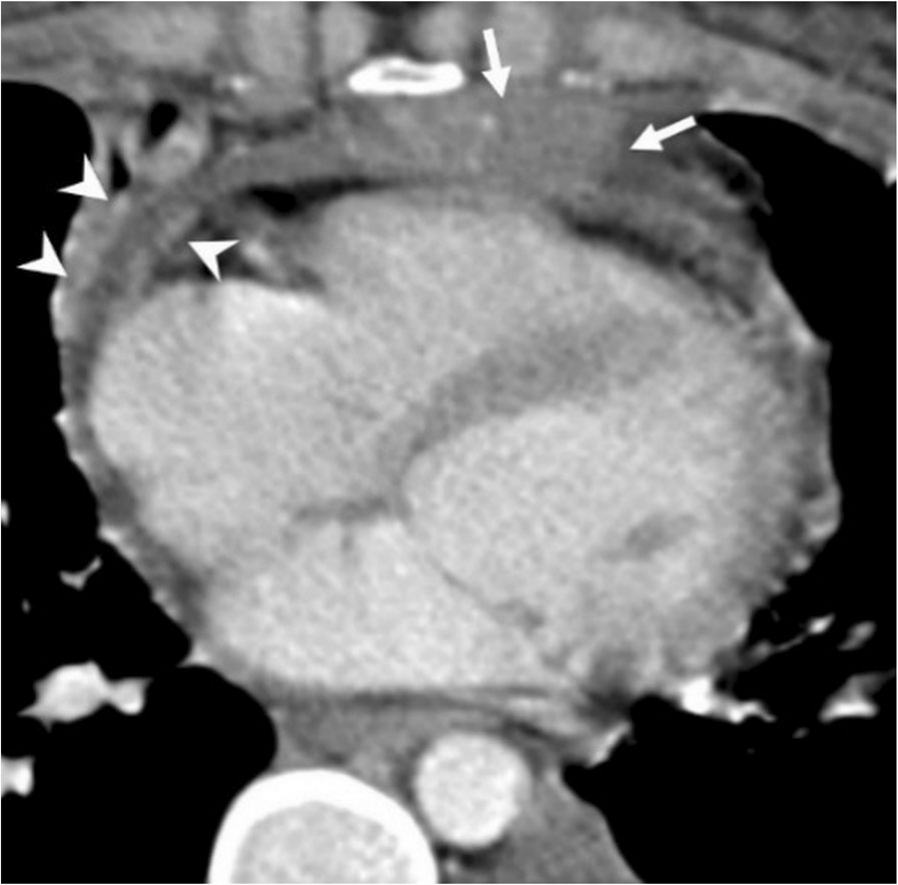
A 26-year-old woman with pathologically proven mediastinal lymphoma. Axial contrast-enhanced CT image demonstrates the inferior aspect of the mediastinal mass (arrows) and pericardial nodular areas of contrast enhancement and thickening suggestive of pericardial involvement (arrowheads)

**Fig. 17:**
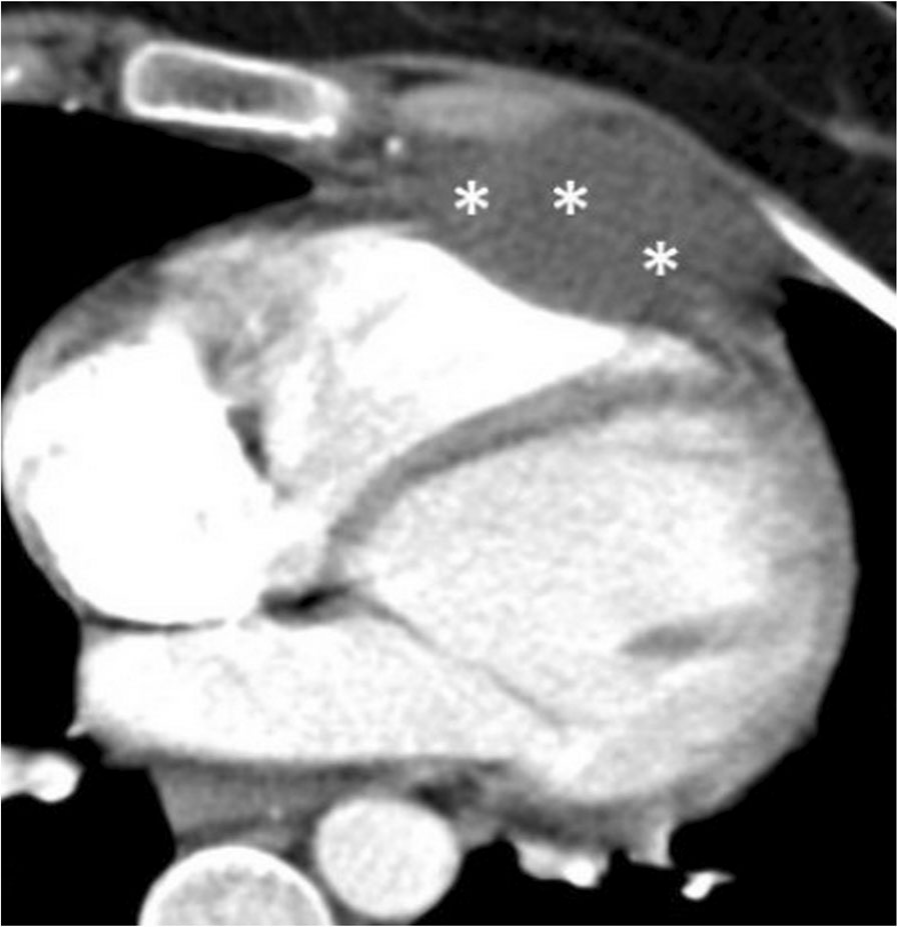
A 28-year-old woman with pathologically proven metastatic osteosarcoma. Axial contrast-enhanced CT image reveals a lobulated hypodense mass (asterisks) invading the anterior aspect of the right ventricle and pericardium

**Fig. 18:**
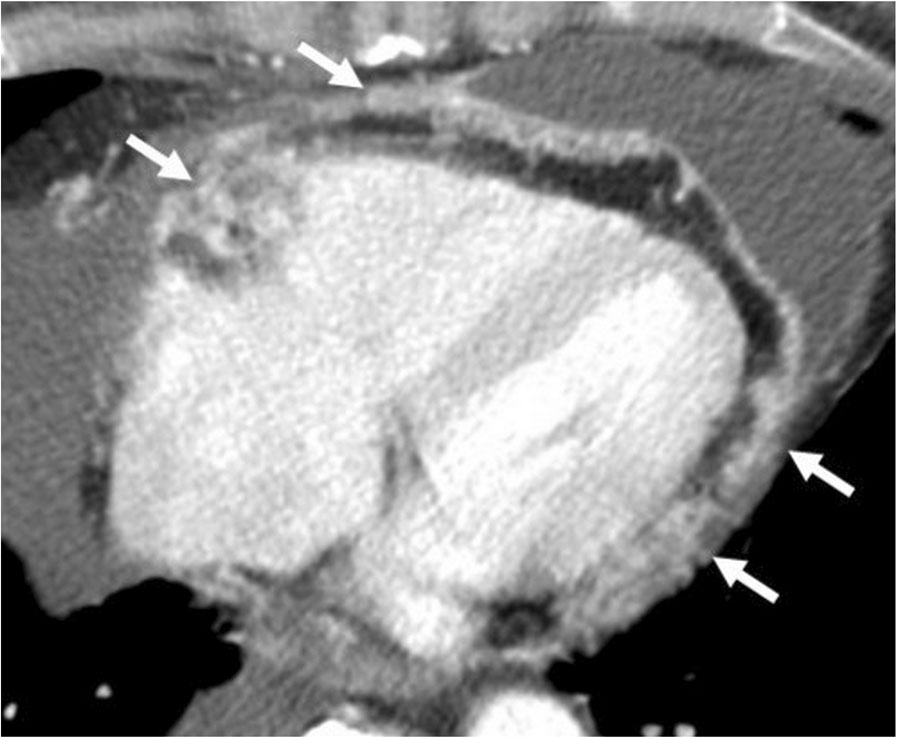
A 48-year-old woman with pathologically proven renal cell carcinoma. Axial contrast-enhanced CT image demonstrates the diffuse irregular thickening and contrast enhancement of the pericardium (arrows) compatible with pericardial metastasis

**Fig. 19:**
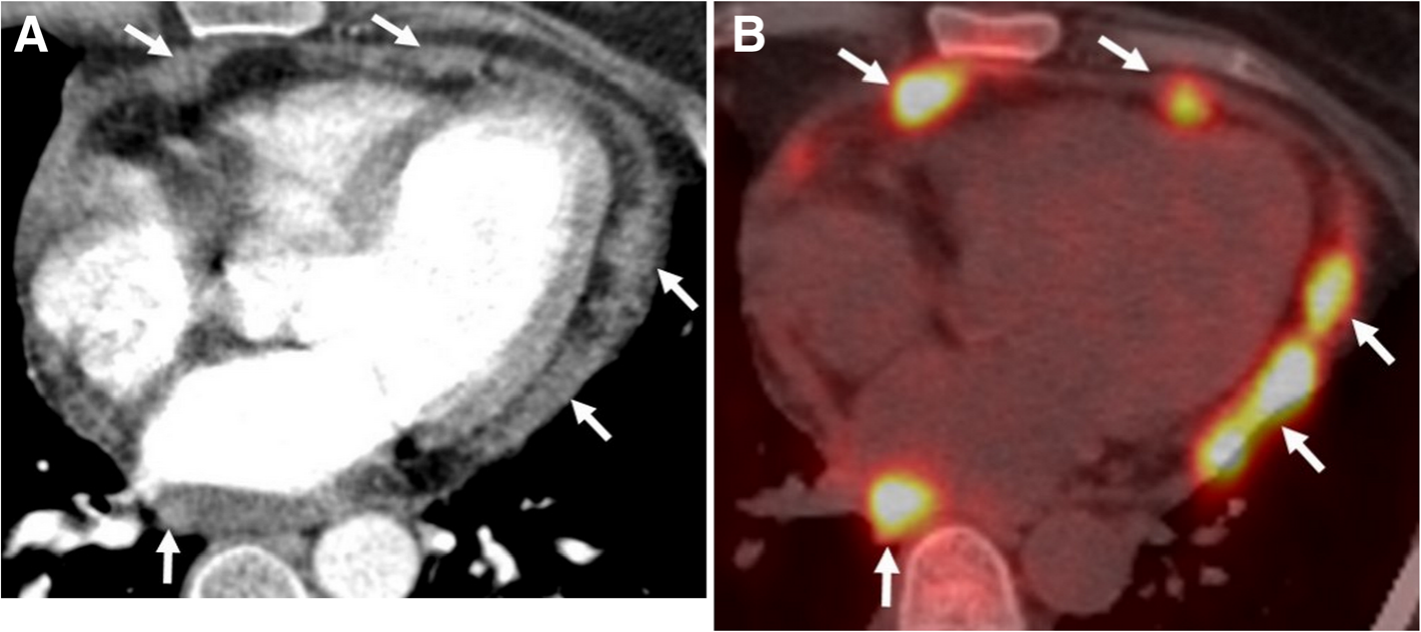
A 55-year-old woman with pathologically proven squamous cell carcinoma metastases from unknown origin. **a** Axial contrast-enhanced CT image demonstrates the nodular areas of pericardial thickening (arrows). **b** PET-CT scan reveals increased FDG uptake (arrows) at the same areas

Pericardial metastases discovered at autopsy are not an uncommon entity among cancer patients [25–27]. Pericardial tumor spread may occur either via hematogenous lymphatic system or direct invasion of an adjacent tumor. Pericardial irregular thickening and/or nodularity, focal, or diffuse FDG uptake and lack of preserved fat plane with an adjacent tumor are the main radiological signs of malignant pericardial involvement (Figs. 15, 16, 17, and 18). Symptoms may vary depending on the severity of involvement. Breast and lung cancers are relatively more common sources of pericardial metastases; however, esophagus cancer, lymphoma, leukemia, melanoma, renal cell carcinoma, and ovarian carcinoma may also metastasize the pericardium [5, 20, 25–28]. Nevertheless, the primary source of metastasis could not be identified in rare cases (Fig. 19).

### Hydatid disease

**Fig. 20:**
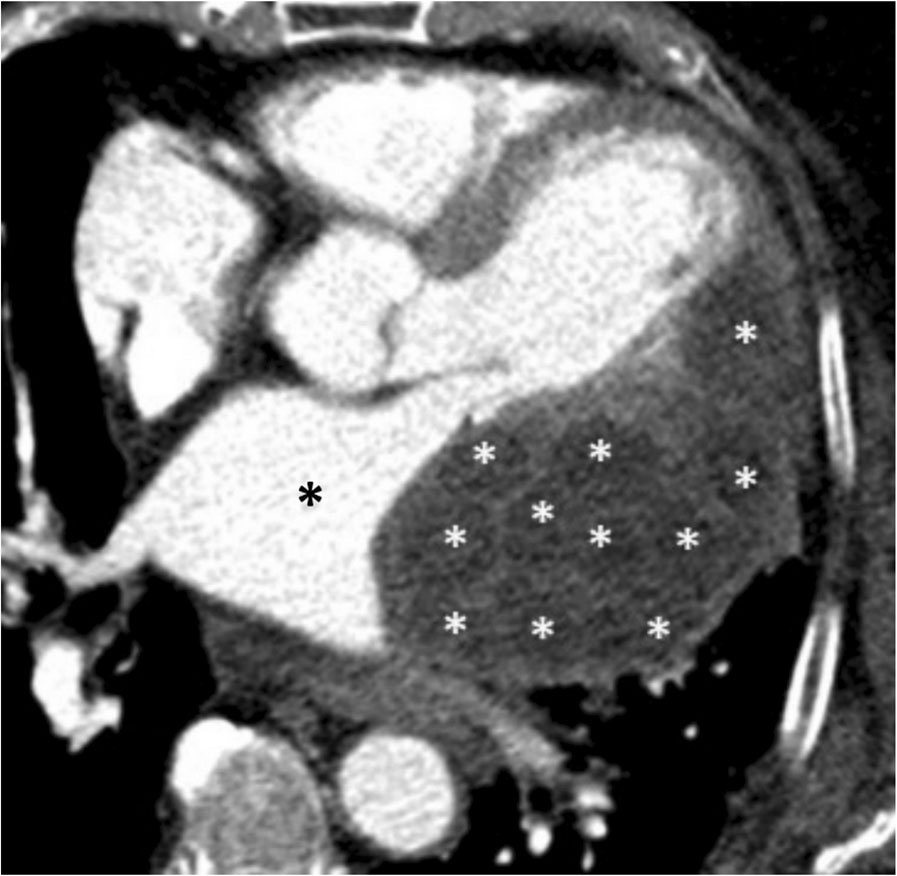
A 86-year-old man with hydatid disease. Axial contrast-enhanced CT image reveals type 3 hydatid cyst containing multiple daughter cysts (white asterisks). Significantly compressed left atrium (black asterisk) due to hydatid cyst abutting the pericardium

Primary pericardial hydatid disease is a rare entity, and it is frequently associated with cardiac hydatid cyst’s rupture into the pericardial space [29]. Presenting symptoms are primarily related to the degree of mass effect to heart chambers. The imaging appearances may vary depending on the degenerative status of hydatid cyst. Presence of the daughter cysts on CT or MRI may give clue for the diagnosis of hydatid cyst (Fig. 20) [30]. Laminated/floating membrane could be more clearly demonstrated by MRI compared to CT [29]. Cysts may compress adjacent heart chambers (Fig. 20). Perforation of the cyst’s content into the pericardial space may result in pericarditis and acute cardiac tamponade [29].

### Medical devices

**Fig. 21:**
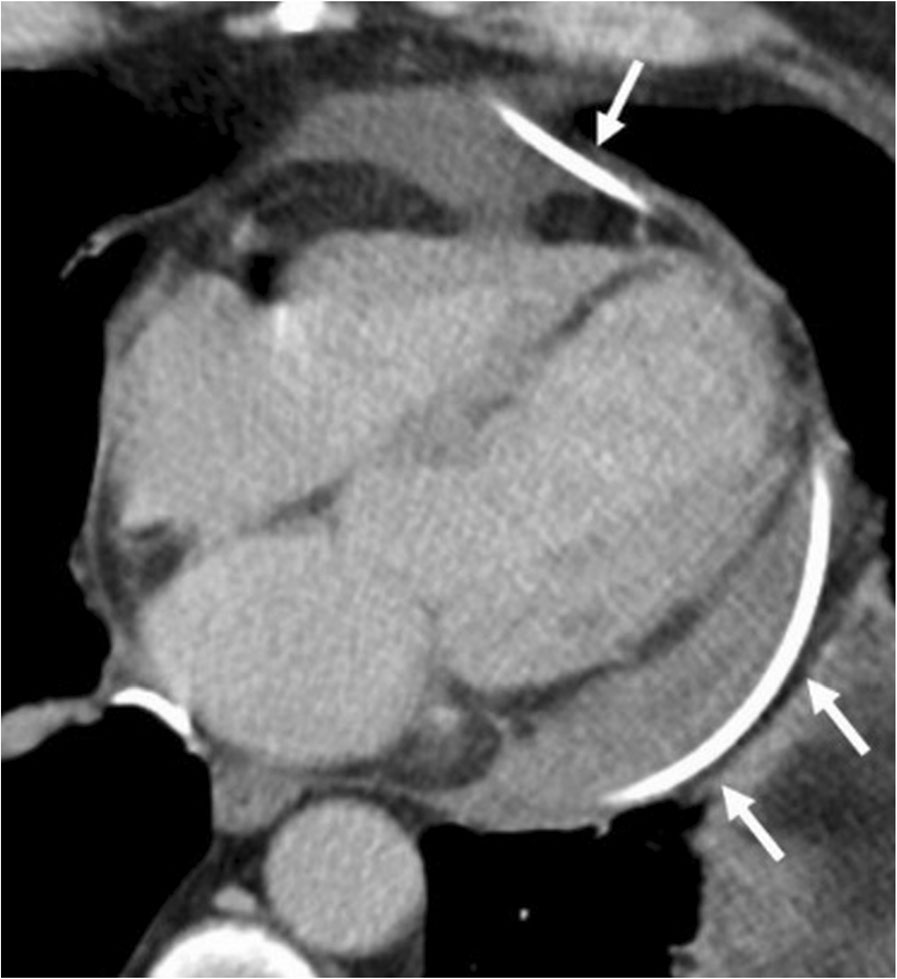
A 80-year-old man with echocardiographic findings suggestive of restrictive physiology due to pericardial effusion. Axial contrast-enhanced CT image demonstrates a drainage catheter coursing within pericardial space (arrows)

Radiologists should be aware of medical devices placed in the pericardial space for certain individual indications. Pericardial drainage catheters are used for therapeutic pericardiocentesis to relieve the pressure applied by pericardial fluid collection to heart chambers. The procedure is performed either echocardiographic or CT guided [2, 31]. A pericardial drainage catheter is seen as a tubular structure coursing within the pericardial space and surrounding heart borders (Fig. 21). In the setting of loculated pericardial effusion, the position of drainage catheter may vary.

**Fig. 22:**
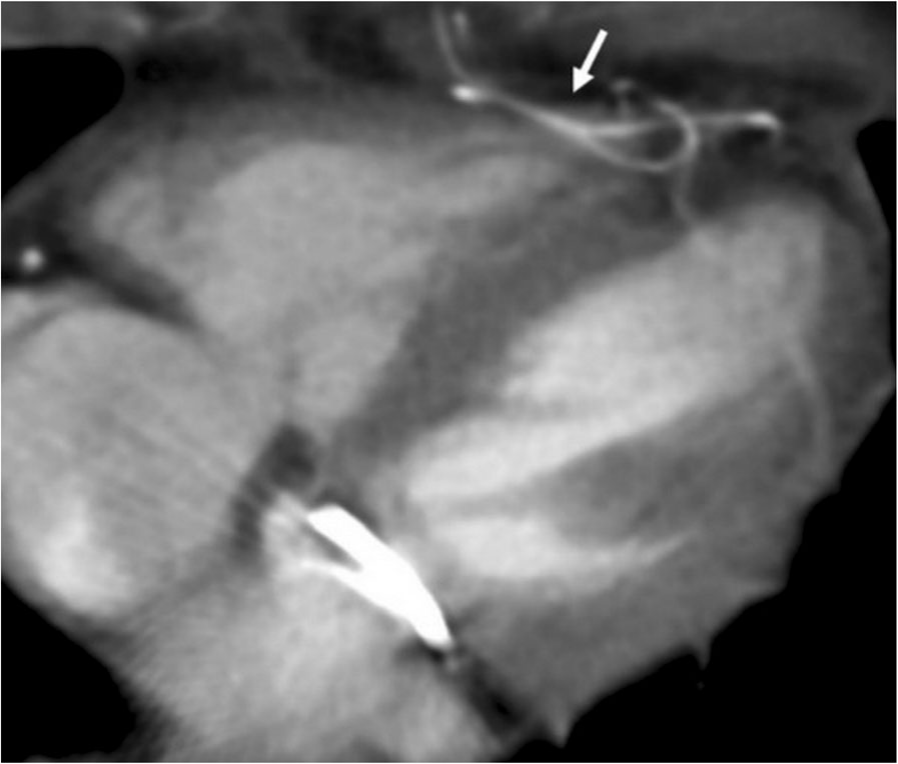
A 45-year-old woman recently underwent mitral valve replacement surgery. Axial contrast-enhanced CT image reveals temporary epicardial pacemaker wires (arrow)

Temporary epicardial pacemaker is used for the treatment of rhythm disturbances seen in the post-operative period following cardiac surgery [32, 33]. Although the wires are not left within the pericardial space, CT may demonstrate wires coursing through the pericardial space before their final attachment to myocardium (Fig. 22).

**Fig. 23:**
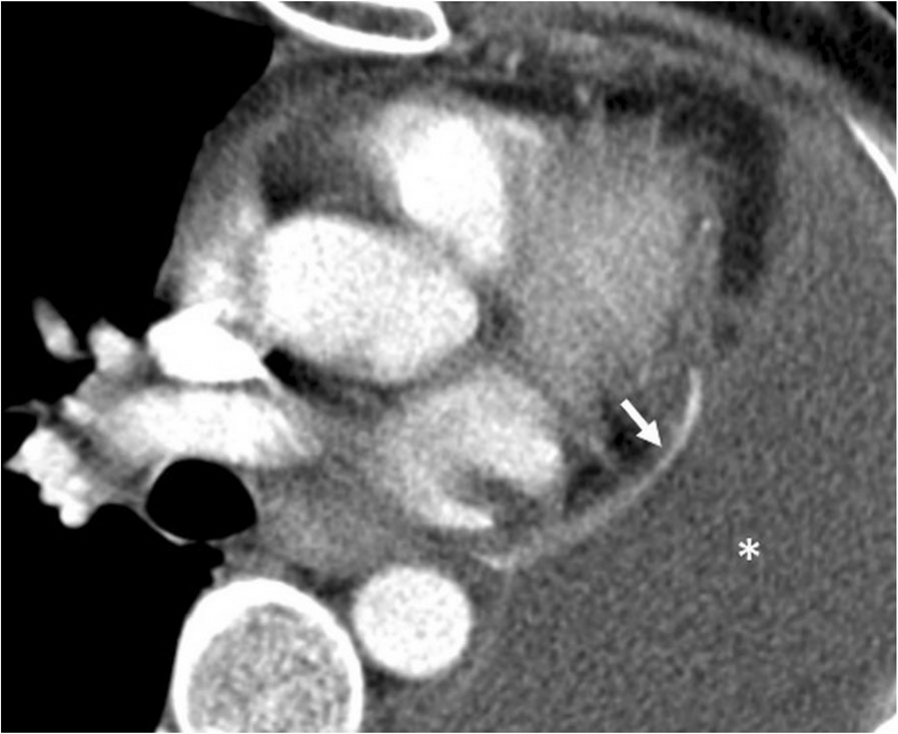
A 50-year-old man with lung cancer. Axial contrast-enhanced CT image demonstrates a linear hyperdense pericardial patch (arrow). Large amount of fluid (asterisk) in left pneumonectomy cavity is also noted

Another medical device that is utilized following surgery is termed as “pericardial patch,” which is primarily used in patients who are at risk for cardiac herniation following extended pneumonectomy with partial pericardiectomy surgery [34–36]. Cardiac herniation is an unfortunate complication, and patients who received induction chemotherapy are reported to be at higher risk. Pericardial defects could be reconstructed using both autologous (pleural flaps, pericardial fat pads, diaphragmatic pedicle flaps, and fascia lata) and synthetic (meshes) materials to avoid cardiac herniation [34–36]. On CT, a pericardial patch could be seen as a hyperdense linear structure supporting the heart border (Fig. 23).

### Fat necrosis

**Fig. 24:**
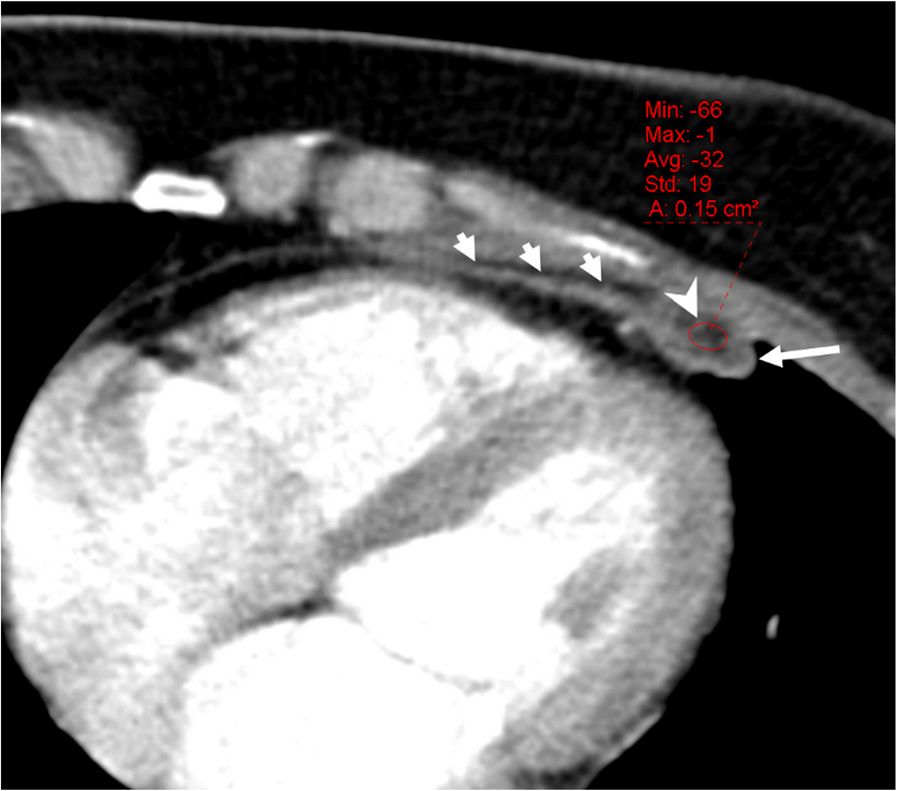
A 46-year-old woman with a 5-day history of chest pain and shortness of breath. Axial contrast-enhanced CT image demonstrates the fat-containing (arrowhead) soft tissue thickness (long arrow) and associated pericardial thickening (short arrows) compatible with pericardial/epipericardial fat necrosis. The CT findings resolved after conservative treatment

Pericardial/epipericardial fat necrosis is a rare, benign, self-limited, and conservatively managed entity with an unknown cause [4, 37]. The CT-based diagnosis is straightforward in most of the cases; therefore, radiologists should recognize the radiologic characteristics of this entity to avoid further examination and unnecessary surgery, since clinical presentation (acute chest pain) may mimic myocardial infarction or pulmonary embolism [4, 37, 38]. The typical CT findings include increasing attenuation of fat tissue adjacent to the pericardium, stranding of the fat, and thickening of the pericardium (Fig. 24). The MRI findings have been reported to be related with the pathologic stages of fat necrosis [39]. Peripheral rim like contrast enhancement (fibrous or granulation tissue) is more apparent after 1 to 5 min following IV administration of gadolinium. Centrally located dark dots and lines on T1- and T2-weighted images have been attributed to the fibrous septa [39]. Subtle inflammation related to fat necrosis could be missed on CT images. However, MRI may better demonstrate the presence of inflammation due to the superior soft tissue contrast resolution.

Epipericardial fat necrosis is reported to occur more frequently on the left side of the hemithorax, and the presence of ipsilateral pleural effusion is not uncommon [38].

### Post-operative changes

**Fig. 25:**
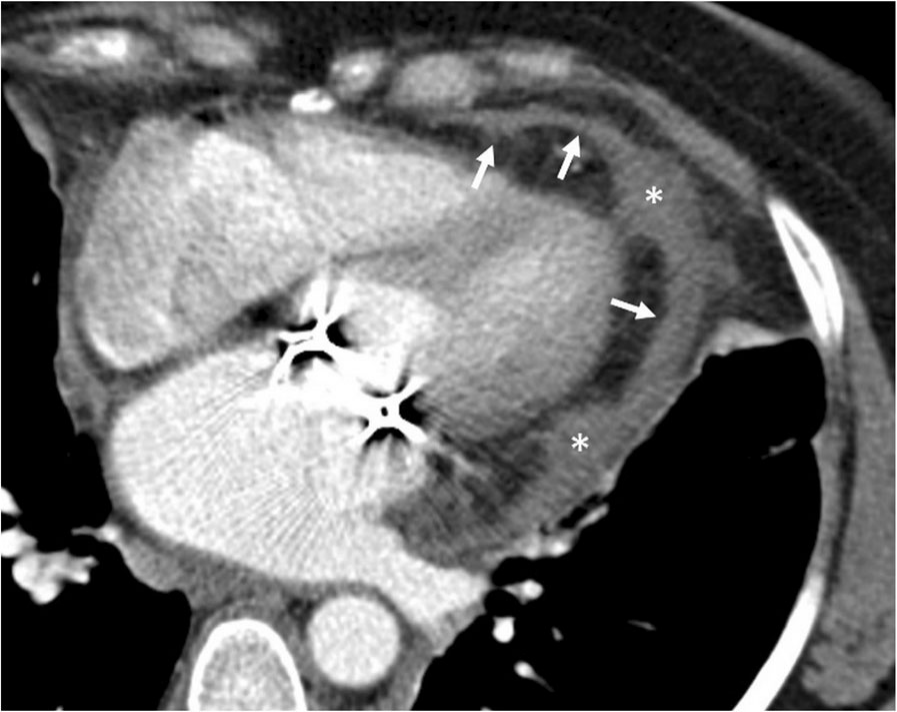
A 49-year-old woman with a previous history of mitral valve replacement surgery. Axial contrast-enhanced CT image shows a pericardial effusion (arrows) with irregular and nodular margins (asterisks). Post-surgical adhesions can mimic imaging signs encountered in pericardial metastasis

**Fig. 26:**
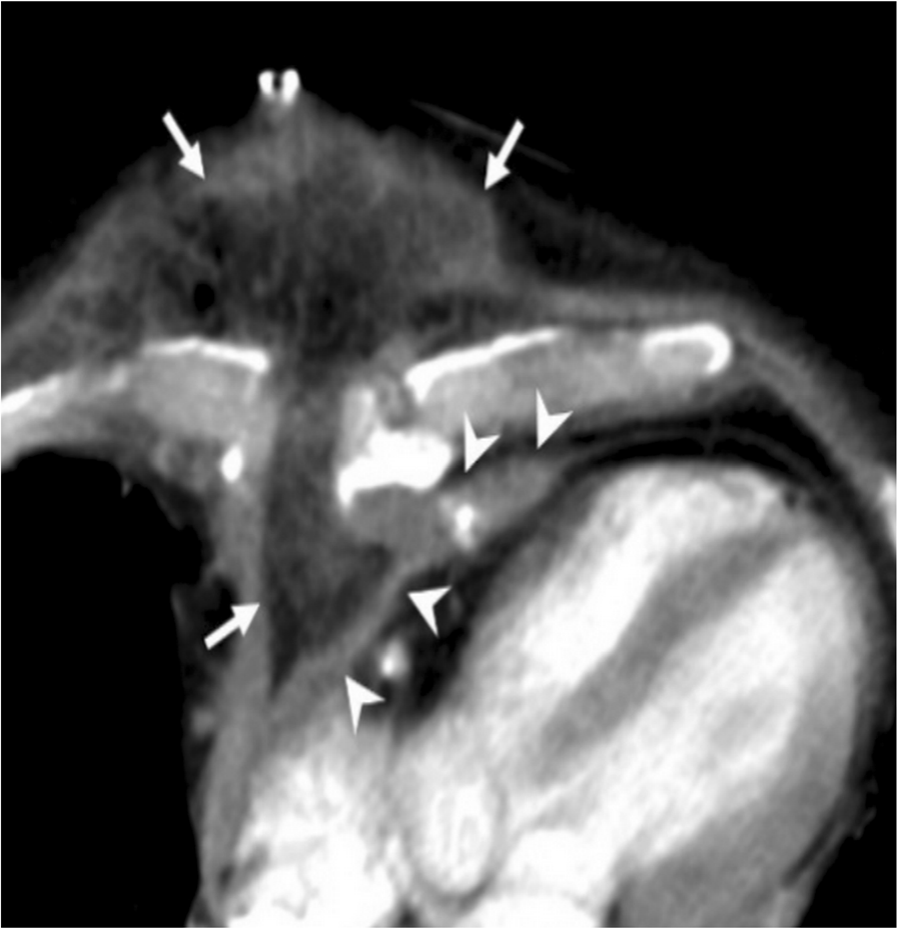
A 65-year-old man with a previous history of surgery for constrictive pericarditis developed chronic sternal osteomyelitis on the follow-up period. Axial contrast-enhanced CT image demonstrates a pedicled fat flap (arrows) placed to maintain hemostasis and infection control. Pericardial thickening (arrowheads) is also noted

Pericardial adhesions which increase the risk of injury to the heart or other major vascular structures during resternotomy could be encountered in the follow-up period of patients who underwent previous cardiac surgery [40]. Pericardial effusion could be seen as areas of nodularity in the setting of adhesions. This appearance may raise suspicion for malignancy due to the irregular pattern of pericardial adhesions (Fig. 25). In addition, a pedicled fat flap applied to maintain hemostasis and infection control could be presented with a fatty pseudo-tumor appearance on CT (Fig. 26) [41].

### Foreign body

**Fig. 27:**
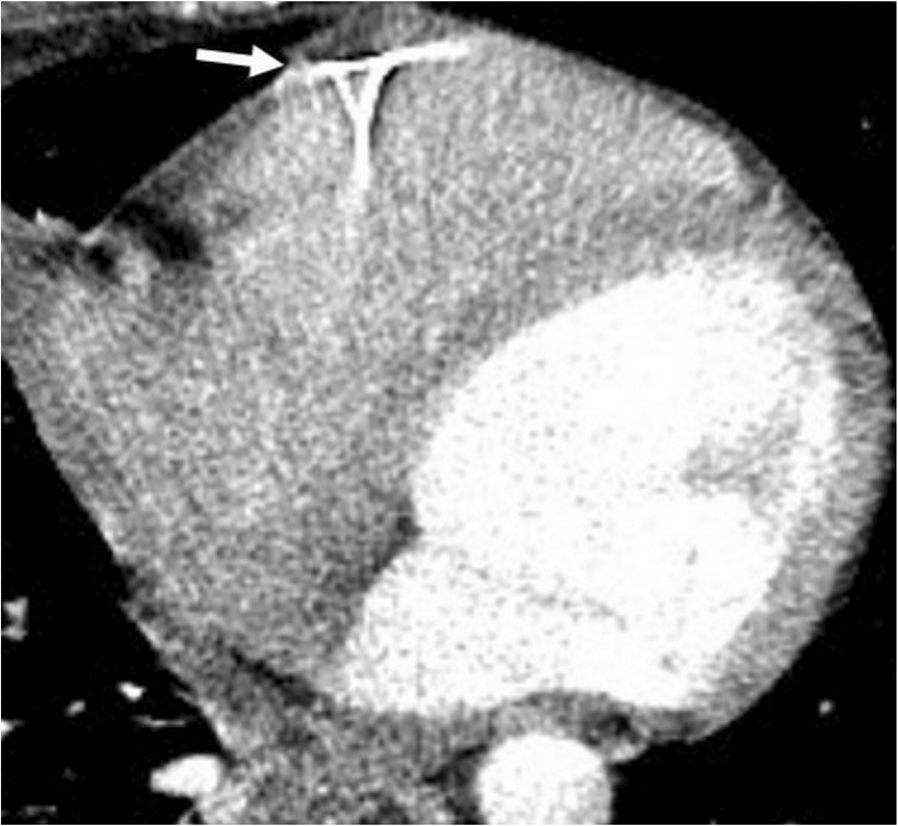
A 41-year-old war veteran. Axial contrast-enhanced CT image demonstrates a shrapnel fragment abutting the pericardium

A foreign body may enter to the pericardial space percutaneously or through the esophagus and central airways [5]. The presence of a foreign body in the pericardium is a rare entity and frequently occurs following trauma (Fig. 27). The imaging appearances may vary due to the nature of the foreign body and type of the insult. The patient could be asymptomatic; however, pericarditis, hemorrhage, tamponade, and cardiac rupture may occur as a complication related to the foreign body [5].

## Conclusion

Imaging findings of pericardial disorders could be non-specific for the majority of the cases; therefore, the patient’s clinical history is the most valuable clue for the differential diagnosis. The radiologists should be familiar with the various imaging appearances of pericardial disorders.
